# Microcirculatory Alterations in Critically Ill Patients with COVID-19-Associated Acute Respiratory Distress Syndrome

**DOI:** 10.3390/jcm11041032

**Published:** 2022-02-16

**Authors:** Umberto Di Dedda, Alice Ascari, Angela Fantinato, Dario Fina, Ekaterina Baryshnikova, Marco Ranucci

**Affiliations:** Department of Cardiovascular Anesthesia and Intensive Care, IRCCS Policlinico San Donato, Via Morandi 30, 20097 San Donato Milanese, Milan, Italy; umberto.didedda@grupposandonato.it (U.D.D.); alice.ascari@yahoo.it (A.A.); angela.fantinato94@gmail.com (A.F.); dario.fina88@gmail.com (D.F.); ekaterina.baryshnikova@gmail.com (E.B.)

**Keywords:** COVID-19, microcirculation, inflammation, endothelial dysfunction, ARDS

## Abstract

Background: Presently, a number of specific observations have been performed on microcirculatory function in a coronavirus disease-19 (COVID-19) setting. We hypothesized that, in the critically ill, endothelial dysfunction secondary to severe acute respiratory syndrome coronavirus 2 (SARS-CoV-2) infection and the subsequent inflammation and coagulopathy may lead to microcirculatory alterations, further exacerbated by the hypoxemic state. A dysfunctional microcirculation may represent the hidden motor underlying the development of COVID-19’s clinical manifestations. Methods: A single center, prospective, observational study. We analyzed bedside sublingual microcirculation in twenty-four consecutive COVID-19-associated acute respiratory distress syndrome (ARDS) patients mechanically ventilated in an Intensive Care Unit (ICU), together with macro-hemodynamics, clinical parameters, echocardiography, and laboratory data at a single time-point after ICU admission. All participants were recruited between March and May 2020. Results: The microcirculatory pattern was characterized by increased values of total vessel density and perfused vessel density, a reduced value of proportion of perfused vessels and microvascular flow index, and high values of heterogeneity index. The duration of mechanical ventilation before microcirculation assessment was inversely associated with the proportion of perfused vessels (*p* = 0.023). Within the macro-hemodynamic parameters, the right ventricle end-diastolic diameter was inversely associated with proportion of perfused vessels and microvascular flow index (*p* = 0.039 and 0.014, respectively) and directly associated with the heterogeneity index (*p* = 0.033). Conclusions: In COVID-19-associated ARDS patients, the microcirculation showed impaired quality of flow parameters coupled with a high vessel density.

## 1. Introduction

The pathophysiology of COVID-19 is still under investigation. Macroscopic clinical features of the SARS-CoV-2 infection, such as the different phenotypes of ARDS or the COVID-19-associated coagulopathy, are relatively well recognized entities, but there is still poor evidence about the micro-level aspects of the disease. COVID-19 is a systemic disease that primarily injures the vascular endothelium, characterized, probably from the early stage, by a disrupted vessel regulation [1].

States of severe inflammation or cardiovascular disease can induce microcirculatory alterations in terms of perfusion patterns, through the damage of cellular sensing mechanisms needed to modulate blood flow [2,3,4,5,6]. The main events underlying microvascular perfusion impairment are inflammation and endothelial activation/dysfunction. Endothelial dysfunction, in particular, is claimed to play a major role through the glycocalyx damage. Glycocalyx breakdown disrupts the natural anti-inflammatory and anti-coagulant properties of the endothelium, and may create a prothrombotic condition triggered by platelet adhesion and aggregation, causing altered local perfusion, functional shunting, and consequent tissue dysoxia [7,8,9,10].

Therefore, microcirculatory perturbations represent a direct link to organ dysfunction.

It is of importance that changes in microvascular perfusion may occur in the absence of global hemodynamic perturbations (i.e., low blood pressure, low cardiac output, hyper-lactatemia), indicating that these alterations are intrinsic to the microcirculation, a condition known as “loss of hemodynamic coherence” [6]. Medical therapies like fluids or vasoactive medications, such as vasopressors, used in states of shock or as reported in COVID-19 ARDS patients [11], can overwhelm endogenous receptor-mediated vessel regulation, further contributing to the loss of hemodynamic coherence. This condition of macro- vs. micro-hemodynamic mismatch is an independent predictor of adverse outcome and organ dysfunction in sepsis and septic shock [7,8,9,10,11,12].

COVID-19 patients exhibit endothelium alterations in microvasculature which has been advocated to be the cornerstone for the development of vasculopathy, altered pulmonary vasoregulation leading to ventilation–perfusion mismatch, and thrombogenesis, with widespread thrombi generation at micro and macrovascular level in the lung and in other organs [13]. These abnormalities have been associated with a higher mortality rate [14,15,16].

To date, a number of studies have investigated the sublingual microcirculation in COVID-19 patients [17,18,19,20,21,22,23] A first evaluation conducted by Damiani et al. [17] in 12 patients with COVID-19 pneumonia showed that sublingual microvascular capillary densities were inversely correlated with D-dimer levels, suggesting the impact of microthrombosis on the microcirculatory function. In a prospective observational study [20], Kanoore Edul et al. demonstrated that sublingual microcirculation in severe COVID-19-ARDS patients was characterized by decreases in PPV (0.96 ± 0.03) and flow quality (MFI: 2.79 ± 0.10 and red blood cell velocity: 1124 ± 161 μm/s) along with high vascular densities (TVD: 21.9 ± 3.9 and PVD: 21.0 ± 3.5 mm/mm^2^), compared to normal values. In a multicenter study including 38 mechanically ventilated COVID-19 patients with moderate-to-severe ARDS [22], Favaron et al. explored the sublingual microcirculation and found that COVID-19 patients showed elevated values of TVD, FCD, capillary hematocrit, capillary-to-systemic hematocrit ratio, and red blood cell velocity in comparison with the microcirculatory parameters of healthy volunteers. In addition, they reported normal values of PPV. Finally, the authors found increased numbers of leukocytes and red blood cell aggregates in the microcirculation, which are likely related to the virus-induced inflammation and hypercoagulability.

A previous report from our group [24], in combination with the other contributions in the literature, suggests the potential role of micro/macro-aggregate formation inside the pulmonary vasculature within the pattern of ARDS in COVID-19 patients.

The purpose of the present study is to assess sublingual microcirculatory status in COVID-19 ARDS patients admitted to ICU under mechanical ventilation (MV), and explore any relation with macrohemodynamics, respiratory function, and laboratory data.

## 2. Materials and Methods

The present study is part of a wide project (COVID-OMICS) prospectively undertaken at the IRCCS Policlinico San Donato at the beginning of the COVID-19 pandemic. The study was performed in line with the principles of Declaration of Helsinki, and approval was granted by the Local Ethics Committee of San Raffaele Hospital (Code: 75/INT/2020) and registered at clinicaltrials.gov (NCT04441502). All the surviving patients gave a written informed consent. We followed the Strengthening the Reporting of Observational studies in Epidemiology (STROBE) checklist for observational studies (https://www.strobestatement.org/fileadmin/Strobe/uploads/checklists/STROBE_checklist_v4_combined.pdf, accessed on 28 December 2021).

### 2.1. Patients and Measurements

This prospective, observational study was conducted in the cardiovascular ICU at the IRCCS Policlinico San Donato, Milan, Italy.

We enrolled twenty-four consecutive patients with laboratory-confirmed COVID-19 and who met the Berlin criteria for ARDS, who were admitted to the ICU between March and May, 2020. All patients were sedated, paralyzed, and ventilated in volume-control mode with Maquet Servo-I ventilators.

The study consisted of a single time-point “photograph” of microcirculatory status, respiratory function, hemodynamics, organ function, biochemical profile, infection, and inflammation indices after the admission into the ICU. Due to the patients’ variable clinical condition at presentation, the measurements were performed soon after ICU admission and oro-tracheal intubation in some cases, and later in others. The median of MV duration before microcirculation assessment was 44 h (IQR: 10–118).

General characteristics of the patient population and the dataset of hemodynamic, ventilatory, respiratory function, laboratory, and clinical parameters are presented in Table 1. Laboratory data of organ function, inflammation, and infection indices were acquired at ICU admission and checked daily. All other parameters were continuously monitored and registered, together with sublingual microcirculation assessment, at the moment of the study. Microcirculatory parameters and timing of sidestream dark field (SDF) measurements of the overall patient population are reported in Table 2. Anticoagulation was established according to a local protocol [24], with an aggressive regimen of low-molecular weight heparin (LMWH): 6000 b.i.d. (8000 IU b.i.d. if body mass index > 35).

The Sequential Organ Failure Assessment (SOFA) score was calculated simultaneously with the study, representing therefore a single time-point image of the degree of organ systems function/dysfunction, coupled with sublingual microcirculatory status.

Microscan (MicroVision Medical, Amsterdam, The Netherlands) was used to obtain sublingual microcirculation data acquiring SDF images.

After carefully removing saliva with gauzes and avoiding suction, the microcirculation was assessed by capturing several consecutive videoclips at different areas of the sublingual mucosa. Prior to analysis, captured videos were selected, four per patient, based on an image quality score (IQS), according to the recommendation by Massey and Shapiro [25].

Each video was subsequently analyzed using the Capillary Mapper 1.4, a recently developed web-based software program (https://capillary-mapper.uni-muenster.de, accessed on 28 December 2021), which enables manual analysis to be carried out according to recommendations of the 2018 Consensus [26].

The Capillary Mapper has been successfully validated for manual analyses of microcirculation videos against the current gold standard, the software AVA 3.2 [27].

For determination of total vessel density (TVD), perfused vessel density (PVD), and proportion of perfused vessels (PPV), microvessels were drawn in by hand using a computer mouse. Capillary flow was then classified as absent, intermittent, sluggish, or continuous. The microvascular flow index (MFI quadrant) was taken as the average of the predominant flow in each of the four quadrants in the video. This, in turn, was used to obtain the heterogeneity index (HI), derived taking into account the highest site flow velocity minus the lowest site flow velocity, divided by the mean flow velocity of all sublingual sites. The present study primarily focuses on capillaries and microvessels (<20 μm in diameter). Manual microcirculation analysis was performed by two external independent examiners, who were blinded to the patient identity and clinical condition.

Transthoracic echocardiography was performed by a single expert operator at the moment of the study in order to assess left and right ventricular dimensions, systolic and diastolic function, valvular status, and estimate systolic pulmonary arterial pressure, according to European Society of Echocardiography guidelines [28].

### 2.2. Endpoints

The primary endpoint was to assess the characteristics of sublingual microcirculatory flow in parallel with global hemodynamic status in COVID-19 associated ARDS patients admitted to ICU.

A secondary endpoint was the identification of differences in microcirculatory function between survivors and non-survivors.

### 2.3. Statistics

Sample size was defined according to the secondary hypothesis. We applied a preliminary hypothesis of a 45% mortality rate, based on the existing Italian and American reports [29,30]. Such mortality rate refers to COVID-19 ARDS patients receiving invasive MV in ICU, namely the most critically ill patient population. By using SDF technology, Edul et al. [31], found that PPV was nearly 88% (standard deviation 10%) in septic shock survivors, and we assumed this value for COVID-19 survivors. We hypothesized that non-survivors had a 15% lower PPV (76%). With an alpha value of 0.05 and a power of 80%, two groups of 12 patients were needed to confirm the experimental hypothesis.

However, at the beginning of the study we could not anticipate (i) the number of patients admitted to our ICU during the pandemic or (ii) the human resources needed to obtain reliable microcirculatory data, and (iii) no data on microcirculation in COVID-19 was available in literature at the time of investigation. Therefore, this sample size was actually settled during the study period.

Data are presented as number (%) or median (interquartile range, IQR). Correlations between continuous variables were assessed using correlation coefficients. For all the tests, a two-tailed *p* value < 0.05 was considered significant. The statistical analyses were conducted using computerized packages (SPPS 13.0, IBM, Chicago, IL, USA, and MedCalc, Ostend, Belgium).

### 2.4. Definitions

Survival was settled at 30-days from ICU admission. Thromboembolic events were defined on the basis of clinically relevant manifestation and by computerized tomography when available.

## 3. Results

Twenty-four patients were included in the study (median age, 63 years [IQR 58–66], 91,7% male). The most common comorbidities were hypertension (37.5%), diabetes (16.7%), and overweightness, with a median body mass index value of 27.7 kg/m^2^ [IQR 24.4–31.2]. Table 1 presents general, clinical, and laboratory characteristics of the patient population at ICU admission.

Twelve patients (50%) died in the ICU, and the others were discharged from the ICU and subsequently from the hospital. Microcirculatory parameters and timing of measurements of the overall patient population are shown in Table 2.

There were some associations between the general clinical profile of the patients and the microcirculatory function. Age was directly associated with TVD (*p* = 0.038) and PVD (*p* = 0.031). None of our series was febrile at the time of the study, nor diagnosed with co-existing bacterial infection. All the patients were intubated and receiving invasive MV before the study for a median period of 44 h [IQR 10–118]. The time under MV before the microcirculatory assessment was inversely associated with PPV (*p* = 0.023). The majority of our patients suffered from moderate ARDS at the time of microcirculation evaluation, with an arterial pO_2_/inspired O_2_ fraction (P/F) median value of 141 ± 48. Median PEEP levels were 12 (11–12) cmH_2_O. At the time of the study, the criteria of protective invasive mechanical ventilation were met.

No association between MV parameters, gas exchange, and microcirculatory variables was found. Patients were hemodynamically stable with nearly normal lactate (1.4 (1.2–1.8) mmol/L) and adequate central venous blood oxygen saturation (78% (74–84)).

No association between coagulation-related parameters (fibrinogen, antithrombin, D-dimer) and microcirculatory variables was found.

Echocardiographic parameters were overall in the normal range, and none of the patients were suffering from cardiac failure or cardiogenic shock at the time of microcirculation assessment. Within the macro-hemodynamic parameters, only the right ventricle end-diastolic diameter (RVEDD) was inversely associated with PPV and MFI (*p* = 0.039 and 0.004, respectively) and directly associated with the HI (*p* = 0.033). The SOFA score was not associated with any of the microcirculation parameters.

Compared to reported normal values [20,31], the microcirculatory pattern was characterized by increased values of TVD and PVD, a reduced value of PPV (<90%), decreased values of MFI below the cut-off value of 2.6 in 17 patients (71%) and high values of HI (Table 2).

In our study, the sample size was probably too small to explore any relationship with the outcome. Hence, the secondary endpoint was not verifiable.

## 4. Discussion

The results of our study may be summarized as follows: (i) mechanically ventilated COVID-19 patients with associated moderate to severe ARDS showed an altered microcirculatory function after admission in the ICU, characterized by an increased capillary density, a low PPV and MFI, and a high HI; and (ii) the main clinical determinants of microcirculation impairment were the time of MV before the examination, and the right ventricular dysfunction expressed as RVEDD.

Determinants of Microcirculatory Alterations in COVID-19 ARDS Patients

Our data show a microcirculatory pattern characterized by supra-normal values of TVD and PVD. Multiple mechanisms may underlie these findings. A normal microvascular density might be linked to the progressive loss of vasomotor tone secondary to the blunted sympathetic drive, as happens in the early phase of septic shock, resulting in capillary vasodilation and preserved microvascular perfusion. In this phase, indeed, the microvascular endothelial response to vasodilating stimuli might be relatively preserved.

As a result of the endothelium invasion by SARS-CoV-2 and the consequent systemic increase in pro-inflammatory cytokines, the infection confers a pro-thrombo-inflammatory phenotype to endothelial cells, eliciting progressive inflammation and systemic vascular thrombosis in macro- and microvasculature, interstitial edema, fibrosis, and architectural rearrangements of microvascular beds, including elongation of venules and sprouting neoangiogenesis [8,32,33]. In our study, we addressed the hemostatic system by measuring the usual clinical parameters, and we could not find an association with microcirculatory changes. However, COVID-19-induced coagulopathy is a complex entity, involving thrombin generation, platelet activation, and fibrinolysis disturbances. It is possible that more sophisticated markers of the hemostatic system may correlate with microcirculatory function [34].

Besides this, severe COVID-19 patients suffer from hypoxemia. The hypoxemic state is a well-recognized powerful trigger for angiogenesis and capillary recruitment [35,36]. Therefore, the reported increased capillary density is likely to be an adaptive response to hypoxemia, a physiologic compensatory reaction to augment the oxygen-extraction capacity by decreasing diffusion distances in the microcirculation. Lastly, microthrombosis is another well-known stimulus for vascular growth [37], accounting for both the increased capillary density and the impaired microcirculatory flow in our series. In this regard, post-mortem studies from COVID-19 patients revealed regular presence of widespread microthrombosis, capillary congestion, and areas of increased capillary density in different organ systems [38,39,40,41].

Our findings are in accordance with recent studies [20,22], while in contrast with others [17,18] reporting decreases in microvascular density in the same setting.

However, our patient population was in more severely hypoxemic conditions (PaO_2_/FiO_2_ 141 ± 48 mmHg) than those reported by the cited authors (PaO_2_/FiO_2_ 207 ± 88 mmHg).

This severe hypoxemic state could represent the predominant factor determining vascular adaptive response towards capillary recruitment and/or neoangiogenesis, thus explaining such differences.

Of importance is that the reported microcirculatory compensatory mechanisms to hypoxemia in COVID-19 disagree with the microcirculatory alterations reported in conventional sepsis. During sepsis, indeed, both the components of microcirculatory oxygen delivery (capillary density and quality of flow parameters) are impaired.

Beside the increased microvascular density, which represents the diffusive component of microcirculatory oxygen delivery, our results showed that the convective components (i.e., MFI, PPV) were impaired.

It has been demonstrated that severe sepsis induces marked alterations of microvascular variables of functional perfusion (PPV, MFI) and of heterogeneity of perfusion (HI), and that such alterations are more severe in the earlier than in the later phase of sepsis [12].

Endothelial dysfunction, and the broad spectrum of its manifestations, is a key mechanism underlying sepsis-induced microvascular alterations.

Accumulating evidence indicates that SARS-CoV-2 infection adversely affects the endothelium of the microcirculation by altering the integrity of vessel barrier, inducing endothelial inflammation and promoting a pro-coagulative state [42,43]. The peculiar hyperinflammatory and pro-coagulant state of COVID-19 implies a critical role of the vascular endothelium for two main reasons: first, the endothelium is the target organ of SARS-CoV-2, and virus entry and proliferation in endothelial cell directly induces damage and apoptosis [44]. Second, the endothelium is the main effector contributing to the inflammatory process and thrombosis. SARS-CoV-2 may cause endothelial dysfunction directly through EC infection, or indirectly through the sustained and exaggerated activation of endothelial cells through a massive pro-inflammatory cytokine release secondary to viral infection [45].

In our COVID-19 series, the reduced blood flow velocity (PPV and MFI) and the increased blood flow heterogeneity (HI) are consistent with the sepsis-induced microcirculatory alterations as described by De Backer et al. [2] and by Edul et al. [31], and are in accordance with a recent study by Rovas et al. [46], where microcirculation was impaired in terms of reduced MFI and PPV in septic patients compared to healthy controls.

In the septic patient, the immediate restoration of a normal microvascular pattern after acetylcholine administration suggests that microthrombi formation is probably not the main determinant of the altered capillary perfusion [2]. Nevertheless, we suspect that in severe COVID-19, as a consequence of the powerful procoagulant state, the widespread microthrombi formation can be tremendous and, thus, may represent the predominant mechanism underlying the impaired microvascular flow in terms of PPV and heterogeneity of perfusion. These changes, indeed, are most likely related to infection-induced thromboinflammation and hypercoagulability.

In parallel, increased HI and reduced PPV reflect the pattern of distributive shock (e.g., sepsis), where vessels plugged by microthrombi are seen next to normally flowing capillaries, representing functional shunting. From a pathophysiological point of view, indeed, the heterogeneity is a key determinant of the shunted fraction, often seen in distributive shock.

The majority of our patients suffered from moderate ARDS at the time of microcirculation assessment. MV-related variables, gas-exchange data, and the lung dysfunction index PaO_2_/FiO_2_ did not show any correlation with the variables of microcirculation.

Interestingly, MV duration before the microcirculation assessment was inversely associated with PPV (*p* = 0.023). Given the inflammatory nature of COVID-19, it might be expected that a longer duration of MV before microcirculatory assessment may be associated with a more impaired microvascular flow, due to the progression of the inflammation process and/or severity of the COVID-19-associated ARDS phenotype.

Of importance is that microcirculatory alterations were not affected by the global hemodynamic state or the use of adrenergic agents, which was limited to two patients only, and at very low doses.

The observed changes in microcirculation function may be therefore intrinsic to the microvasculature, occurring in the absence of macro-hemodynamic perturbations, as shown in our series, defining a scenario of loss of hemodynamic coherence.

Not surprisingly, indeed, other perfusion-related macrohemodynamic parameters such as lactate, capillary refill time, and central venous oxygen saturation were, on average, in the normal range at the time of microcirculation assessment.

It must be stressed that the aim of the present study was the assessment of microcirculatory function restricted to the early phase of the disease. We did not perform sequential sublingual microcirculation assessments in the more advanced state of the disease. As microcirculation is a dynamic process, more significant microcirculatory abnormalities, compared with baseline, might have then been identified, possibly bringing out any association with improvement or decline of organ function over time.

The impaired microcirculation parameters that we found in our series are then probably to be ascribed to the effects of more aggressive forms of COVID-19, coupled with preexisting patients’ individual factors such as cardiovascular disease, diabetes, or obesity, namely conditions of pre-existing endothelial dysfunction. In these cases, apparently, the deleterious effects of inflammation and hypoxia are precociously visible and detectable even at microvascular level.

Regarding the hypothesized prognostic power of microcirculation function in this new setting, we did not find any statistically significant correlation with the SOFA score.

COVID-19 can also cause a wide range of cardiac injury including acute myocardial infarction, stress-related cardiomyopathy, myocarditis, nonischemic myocardial injury due to hyperinflammatory cytokine storm, and microvascular dysfunction affecting the left or right ventricle (RV) [44,47]. We suspect that the formation of pulmonary microthrombi increases the risk of subsequent RV dysfunction, explaining our finding of impaired RV function in terms of RVEDD in 11 (46%) patients.

Indeed, abnormal RVEDD (>34 mm) was inversely associated with quality of flow in the sublingual microcirculation (PPV and MFI) and, accordingly, directly associated with an increased heterogeneity of perfusion (HI).

No-one in this series was diagnosed with cardiogenic shock at the time of microcirculation assessment. Nevertheless, similar microvascular alterations have been observed in patients with severe heart failure and are more severe in non-survivors [48,49].

The findings of this study have to be seen in light of some limitations. Due to the peculiar characteristics of this pandemic disease, the complex technology, and the limited human resources, we could include only a limited series of patients. Our sample size is however comparable to what already published.

Furthermore, measurements were taken at a single time point, during the acute phase of a changing clinical course, which does not exclude possible different findings in other stages of the illness. As microcirculation is a dynamic process, further investigation of the microcirculatory function trend, rather than an instant picture, might highlight the progressive changes of microcirculatory function in COVID-19’s clinical evolution.

In conclusion, our results show that in moderate to severe COVID-19 ARDS patients, the microcirculation is disturbed in terms of density, perfusion, and flow heterogeneity. The vascular endothelial dysfunction secondary to SARS-CoV-2 infection results in widespread inflammation and coagulopathy, which, together with hypoxemia, lead to various degrees of dysfunction in the microvasculature.

We suspect that, as happens in sepsis, microcirculatory alterations represent the motor of the cascade of events leading to organ dysfunction and bad outcomes. Therefore, besides a solid knowledge of what happens in septic patients, a compelling need for microcirculation monitoring at the bedside in COVID-19 ICU patients is advocated.

## Figures and Tables

**Table 1:** General characteristics of the patient population (N = 24).

Variable	Measure
Age (years)	63 (58–66)
Gender male	22 (91.7)
Weight (kgs)	85 (76–94)
Body mass index (kg/m^2^)	27.7 (24.4–31.2)
Mechanical ventilation before microcirculation assessment (hours)	44 (10–118)
Hypertension	9 (37.5)
Diabetes	4 (16.7)
Smoking history	2 (8.3)
Chronic obstructive pulmonary disease	2 (8.3)
Cardiovascular disease	3 (12.5)
Chronic kidney failure	1 (4.2)
Tympanic temperature (°C)	36.5 (36.2–36.9)
Drugs at microcirculation assessment	
Propofol	23 (95.8)
Midazolam	1 (4.2)
Remifentanil	23 (95.8)
Dexmedetomidine	2 (8.3)
Anti-viral agents	8 (33.3)
Antibiotics	12 (50)
Tocilizumab	8 (33.3)
Steroids	20 (83.3)
Anti-thrombotic drugs at microcirculation assessment	
Low molecular weight heparin	24 (100)
P_2_Y_12_ inhibitors	11 (45.8)
Vasoactive drugs	
Nor-epinephrine	3 (12.5)
Epinephrine	2 (8.3)
Dopamine	2 (8.3)
Macro-hemodynamic pattern	
Mean arterial pressure (mmHg)	95 (81–107)
Heart rate (beats/minute)	82 (58–102)
Central venous pressure (mmHg)	11 (9–13)
Echocardiographic parameters	
Left ventricular ejection fraction (%)	52 (45–65)
Right ventricular end-diastolic diameter (mm)	34 (28–38)
Tricuspid annular plane systolic excursion (mm)	20 (18–20)
Systolic pulmonary arterial pressure (mmHg)	30 (25–31)
Mechanical ventilation and gas exchange	
Tidal volume (mL)	500 (462–557)
Respiratory rate (cycles/min)	20 (16–22)
Inspired oxygen fraction	0.65 (0.60–0.84)
Positive end expiratory pressure (cmH_2_O)	12 (11–12)
Plateau pressure (cm/H_2_O)	24 (22–26)
Driving pressure (cm/H_2_O)	12 (11–13)
Arterial blood pH	7.39 (7.34–7.45)
Arterial blood pCO_2_, mmHg	44 (41–54)
Arterial blood pO_2_, mmHg	91 (73–98)
Arterial blood oxygen saturation (%)	96 (94–97)
Arterial pO_2_/Inspired O_2_ fraction	141 ± 48
Central venous blood oxygen saturation (%)	78 (74–84)
Arterial blood lactates (mmol/L)	1.4 (1.2–1.8)
Laboratory values	
White blood cells (×1000/μL)	8.8 (6.3–12.3)
Hemoglobin (g/dL)	11 (9.8–12.2)
Platelets (×1000/μL)	264 (155–391)
Serum bilirubin (mg/dL)	0.53 (0.39–0.87)
Serum creatinine (mg/dL)	0.98 (0.70–1.15)
Albumin (mg/dL)	2.3 (2.1–2.8)
Interleukin-6 (pg/mL)	112 (50–266)
Prothrombin time (seconds)	87 (81–89)
Activated partial thromboplastin time (seconds)	35 (30–41)
Fibrinogen (mg/dL)	770 (623–873)
Antithrombin activity (%)	92 (82–103)
D-Dimers (μg/mL)	2.4 (1.2–3.8)
SOFA score (points)	4 (3–4.75)

Data are number (%) or median (interquartile range); SOFA: Sequential Organ Failure Assessment.

**Table 2:** Microcirculatory parameters (compared to normal values) and timing of SDF measurements in the overall patient population (N = 24).

Microcirculatory Parameters	Value, Median	Value, Mean	Normal Values	*p*-Value
Total vessel density (mm/mm^2^)	24.8 (21.6–25.8)	23,825 ± 3.03	16.7 ± 1.6	<0.0001
Perfused vessel density (mm/mm^2^)	21.6 (18.7–23.5)	21.188 ± 3.6	16.6 ± 1.6	<0.0001
Percentage of perfused vessels (%)	88.8 (86.2–95.3)	89.815 ± 6.9	100.00 ± 0.0	<0.0001
Microvascular flow index (points)	2.43 (2.31–2.69)	2.457 ± 0.315	2.97 ± 0.03	<0.0001
Heterogeneity index (arbitrary units)	0.41 (0.28–0.45)	0.381 ± 0.148	0.04 ± 0.03	<0.0001
Onset of symptoms to exam (days)	12.5 (9.2–15.8)			
Tracheal intubation to exam (hours)	44 (10.2–118)			

Data are median (interquartile range) and mean (standard deviation); SDF: Sidestream Dark Field. For normal values of microcirculatory parameters see [20].

## Data Availability

The datasets generated and/or analyzed during the current study are available from the corresponding author on reasonable request.
